# Building an Extracorporeal Cardiopulmonary Resuscitation Program at a High-volume Extracorporeal Membrane Oxygenation Center

**DOI:** 10.1051/ject/2023042

**Published:** 2023-12-15

**Authors:** Peter C Michalakes, Walter F DeNino, Claire B Jara, Maxwell E Afari, Bram J Geller

**Affiliations:** 1 Tufts University School of Medicine Boston MA 02111 USA; 2 Department of Cardiac Surgery, Maine Medical Center Portland ME 04102 USA; 3 Department of Cardiovascular Perfusion, Maine Medical Center Portland ME 04102 USA; 4 Department of Advanced Heart Failure and Transplant Cardiology, Maine Medical Center Portland ME 04102 USA; 5 Department of Cardiovascular Medicine, and Cardiovascular Critical Care Services, Maine Medical Center Portland ME 04102 USA

**Keywords:** Extracorporeal Cardiopulmonary Resuscitation (ECPR), Extracorporeal Membrane Oxygenation (ECMO), Cardiac arrest

## Abstract

Extracorporeal Cardiopulmonary Resuscitation (ECPR) is an emerging approach to cardiac arrest. We present two contrasting cases from a high-volume extracorporeal membrane oxygenation (ECMO) center (defined as greater than 30 ECMO cases per year) without a 24/7 ECPR program to highlight how to establish an ECPR program with a focus on patient selection and outcome optimization. In one case, a patient presented with cardiac arrest during initial triage for chest pain within the emergency department, and in the other case, a patient experienced an out-of-hospital cardiac arrest with prolonged no-flow and low-flow time. Despite the lack of a 24/7 ECPR program at the presenting center, both patients received an ECPR evaluation, as both patients presented while all services necessary for ECMO cannulation were available. The in-hospital cardiac arrest patient was successfully cannulated for ECMO during cardiopulmonary resuscitation and survived with few complications. The out-of-hospital cardiac arrest patient was deemed a poor candidate for ECPR and expired soon after presentation. These two cases highlight the complex decision-making in ECPR and further illustrate how to create ECPR protocols at a high-volume ECMO center before resources are available for a 24/7 ECPR program.

## Overview

Extracorporeal cardiopulmonary resuscitation (ECPR) is an emerging approach for the management of both in-hospital cardiac arrest (IHCA) and out-of-hospital cardiac arrest (OHCA). Current evidence for ECPR is challenging to interpret given various selection criteria and protocol variations [1]. The ARREST trial in 2020 was a single-center randomized trial of patients with ventricular tachycardia/ventricular fibrillation (VT/VF) OHCA who were randomized to ECPR or standard of care. The trial was terminated early due to a significant survival benefit to ECPR [2]. Subsequently, a single-center randomized trial in Prague did not show a mortality benefit in OHCA from a presumed cardiac cause despite a bundle of care involving intra-arrest transport, ECPR, and immediate invasive assessment and treatment. The aforementioned study was stopped early due to futility, though the study was performed as an intention-to-treat analysis and a substantial percentage of patients had a return of spontaneous circulation (ROSC) after randomization but prior to hospital arrival [3]. A secondary analysis of this trial found improved survival with ECPR for OHCA when patients who achieved ROSC before hospital arrival were separated from the standard of care and intervention groups [4]. Most recently, in 2023, the INCEPTION trial found no significant differences in survival between ECPR and standard treatment for OHCA [5], lending greater uncertainty to the role of ECPR for OHCA. However, like the Prague trial, many patients in the INCEPTION trial also had ROSC after randomization and before hospital arrival. Notably, the ARREST and INCEPTION trials included patients only with shockable rhythms [2, 5, 6].

A meta-analysis in 2023 assessing ECPR for OHCA, including the above trials, confirmed the benefit of ECPR [6]. In this meta-analysis, while ECPR increased survival compared to conventional CPR regardless of the rhythm, there was a greater benefit in patients with an initial shockable rhythm [6]. The methodology in these clinical trials has an important impact on their findings; as a result, despite conflicting data, many providers still feel ECPR has a role in appropriately selecting patients.

Consequently, many centers are expanding their extracorporeal membrane oxygenation (ECMO) programs to include ECPR. However, given the resource-intensive nature of ECPR, including the requirement for 24/7 availability of both ECMO specialists and cannulating physicians, and ongoing uncertainty regarding the best ways to implement ECPR, the growth of ECPR has been variable. Many ECMO centers do not have established ECPR programs, as current literature is inconsistent, resource allocation is significant, bed availability is often limited, and staffing may be insufficient to support such a program. Despite these limitations and uncertainty, we propose that high-volume ECMO centers should consider establishing a limited ECPR program based on local cardiac-arrest care needs using the strategy implemented at our center even in the absence of pre-existing 24/7 cannulation capacity.

## Description

We propose three specific recommendations for ECMO programs as they seek to develop an ECPR program. First, it is most effective to focus ECPR development at centers with larger ECMO volumes, since adult ECMO patients at centers with greater than 30 annual ECMO cases have been shown to have improved survival rates [7]. Furthermore, a recent analysis of the Extracorporeal Life Support Organization Registry found a possible survival benefit among ECPR patients who received ECPR at centers with greater than 12 ECPR cases per year [8]. We recommend that the development of ECPR programs be focused at high-volume centers (those centers with greater than 30 ECMO cases per year), such as our center.

Second, given the inherent logistical challenges in offering ECPR, we propose that high-volume ECMO centers can begin building an ECPR program by offering ECPR when ECMO specialists and the cannulation team are generally already in-house, such as during regular daytime hours. This “part-time” approach to ECPR delivery allows institutions to carefully select cases and begin to develop the protocols and infrastructure to support such patients. Such infrastructure could then be used to justify the resources and staffing required to create a comprehensive, 24/7 ECPR program.

Third, it is crucial for every ECPR program to have precise selection criteria and protocols. There is currently insufficient evidence to establish universal selection criteria for ECPR [1], however, certain principles do exist that can inform initial program development, which are summarized in Table 1. Here, we present two cases considered for ECPR that we believe reflect these principles and demonstrate that offering such a service is both realistic and beneficial to select patients.

**Table 1:** Suggested selection criteria for new ECPR programs.

No-flow time	5 min or less
Low-flow time	60 min or less
Witnessed and unwitnessed arrests	Witnessed arrests with bystander CPR
Age	Under 60/65 years
Initial cardiac rhythm	Shockable rhythms

### Case 1: In-Hospital Cardiac Arrest (IHCA)

A male patient in his 50s with a history of hypertension presented to the emergency department with chest pain. During triage, the patient went into VF, and Advanced Cardiovascular Life Support (ACLS) was initiated. ROSC could not be obtained despite nearly 15 attempts at defibrillation and ongoing CPR, and the patient was emergently placed on peripheral veno-arterial (VA)-ECMO. He ultimately received approximately 50 min of CPR prior to achieving full VA-ECMO support. The patient went emergently to the catheterization laboratory, where he was found to have an occluded proximal left anterior descending artery (LAD). In the catheterization lab, a drug-eluting stent was placed in the LAD and an Impella CP was placed for left ventricular unloading. Immediately after revascularization, he was successfully defibrillated into normal sinus rhythm. The patient underwent targeted temperature management to 36 °C for 24 h.

His overall hospital course was complicated by cardiogenic shock, the placement of a semi-permanent pacemaker due to persistent bradycardia, hypoxic-ischemic encephalopathy, agitation, pneumonia, and tracheostomy placement in the setting of acute respiratory failure. After approximately 72 h of support, both the Impella CP device and VA-ECMO circuit were removed.

His tracheostomy site was decannulated and he was discharged on hospital day 33. At follow-up, he is fully independent in activities of daily living, driving, and has returned to work consistent with a Cerebral Performance Category of 1.

### Case 2: Out-of-Hospital Cardiac Arrest (OHCA)

A female patient in her 20s, G1P0 at 7 weeks gestation, with a history of pulmonary embolism and a hypercoagulable state, was prescribed enoxaparin sodium for anticoagulation, but due to insurance issues had a lapse in adherence. She presented emergently by ambulance after being found down. Emergency Medical Services (EMS) initially reported a bradycardic rhythm, but during transport, she became pulseless. An ACLS response for pulseless electrical activity arrest was initiated and a LUCAS device was placed for mechanical chest compressions. Unfortunately, due to the rural location of the patient, the time between her initial pulselessness and her arrival at the ECMO center was prolonged to approximately 60 min.

A shock team consult was emergently performed. On further history, the patient may have had approximately 20 min of cardiac arrest without bystander CPR before pulselessness was recognized. She then had CPR for an additional 40 min. Ultrasound evaluation showed thrombosed femoral arteries bilaterally and the decision was made to end all resuscitative measures and the patient expired.

## Discussion

These two cases highlight key selection criteria and features of developing a successful ECPR program. In developing an ECPR program, high-volume ECMO centers can begin to offer ECPR assessments during the hours when ECMO specialists (such as perfusionists) and cannulating physicians are already in-house, as in these cases. By offering ECPR services during selective hours, high-volume ECMO centers can begin to establish positive outcomes and the infrastructure required for ECPR. At our center, ECPR evaluations are offered during weekday daytime hours, which is when both perfusionists and cannulating physicians are in-house. Staffing has been organized to ensure a cannulating physician is always available outside of the operating room during this period to provide these services. To facilitate greater utilization of ECPR evaluations, we have publicized the availability of this service to hospital staff, and we encourage clinicians to contact the ECPR team early even if there is uncertainty regarding the patient’s candidacy for ECPR. We recommend teams call for an ECPR evaluation within 5 min of cardiac arrest for any patient without clear contraindications to ECPR.

When considering staffing for a new ECPR team, we recommend including an intensivist, perfusionist, cannulating physician (typically a cardiac surgeon), and a cardiologist (either a critical care cardiologist or a heart failure cardiologist). These roles may overlap with preexisting cardiogenic shock teams, and combining these two teams is an efficient method of staffing a new ECPR program. When considering bed and ECMO capacity for a new program, we suspect that annual ECMO volume is an effective indicator of readiness to start a new ECPR program, as high annual ECMO volume implies capacity and familiarity to treat this population of patients.

In general, it would be advisable to have at least three ECMO circuits available prior to starting a part-time ECPR program, to ensure capacity for at least two simultaneous patients and one backup circuit. For those ECMO centers without cardiogenic shock teams, the development of a new ECPR program should occur in concert with other efforts to improve the care of patients who require any form of temporary mechanical circulatory support. Once established, a successful part-time ECPR program can look to measures of ECPR volume to assess readiness for a full-time ECPR program, as a high monthly volume of ECPR cases may indicate a sufficiently high case volume to justify full-time ECPR services.

To improve ECPR outcomes, several factors should be carefully considered during the assessment process and built into protocols (Table 1): time without CPR (“no-flow” time), time with CPR (“low-flow” time), shockable rhythm, age, and witnessed collapse [9]. The importance of no-flow time is reflected in these two cases, as the IHCA patient experienced extremely short no-flow time, while in the OHCA case, no-flow time was prolonged. In cases where no-flow time is clearly discernible, we recommend including upper limits on no-flow time, such as 5 min [9]. Other centers have made a witnessed cardiac arrest with bystander CPR a requirement for ECPR consideration, which is a reasonable requirement for nascent programs building protocols and optimizing outcomes [10]. Similarly, limiting low-flow time to under 60 min has been associated with positive ECPR outcomes [9]. In cases of OHCA, close coordination with EMS is required when developing ECPR programs to ensure appropriate patient selection and rapid transfer of care. Given evidence of improved outcomes at centers with dedicated post-cardiac arrest services, it is crucial to coordinate the transport of patients quickly to such a center [10].

Finally, these two cases highlight the importance of integrating shock and cannulation teams early. We suggest that ECPR teams should be notified of potential OHCA patients prior to patient arrival if feasible. Notifying the ECPR team after initial resuscitative efforts are unsuccessful introduces significant delays in ECPR that can negatively affect outcomes, and we suggest early evaluation, such as within 5–10 min of CPR initiation, even if the patient ultimately achieves ROSC and does not require ECPR. This is a particularly useful approach in the setting of reversible causes of cardiac arrest, as demonstrated in the IHCA case. Regarding other potential prognostic factors (such as age and shockable rhythm) [9], centers elsewhere have incorporated these factors into their ECPR protocols. Early ECPR programs may choose to focus a program on patients under the age of 60/65 years old and those with a shockable rhythm [10]. These protocols should be continuously modified to reflect both institutional trends and the most recent evidence regarding ECPR.

These two cases serve as illustrative examples of how a high-volume ECMO center can start an ECPR program. In summary, we recommend building ECPR programs at high-volume ECMO centers; such programs may initially exist as part-time programs with strict patient selection criteria, with the anticipation of program evolution as the literature in this space develops. Through this process, ECMO centers can develop protocols, local expertise, and the infrastructure needed to care for these complex patients. Over time, through a successful “part-time” ECPR program, institutional support may develop to support fully comprehensive ECPR services that can evolve in concert with changing clinical evidence.

## Data Availability

All data are included or referenced within the article.
